# Antifungal Activity and Chemical Composition of Seven Essential Oils to Control the Main Seedborne Fungi of Cucurbits

**DOI:** 10.3390/antibiotics10020104

**Published:** 2021-01-22

**Authors:** Marwa Moumni, Gianfranco Romanazzi, Basma Najar, Luisa Pistelli, Hajer Ben Amara, Kaies Mezrioui, Olfa Karous, Ikbal Chaieb, Mohamed Bechir Allagui

**Affiliations:** 1 Laboratory of Plant Protection, National Institute for Agronomic Research of Tunisia, University of Carthage, Rue Hédi Karray, Ariana 2080, Tunisia; m.moumni@staff.univpm.it (M.M.); mzbenamara@gmail.com (H.B.A.); s1087850@studenti.univpm.it (K.M.); allagui.bechir@gmail.com (M.B.A.); 2 Department of Agricultural, Food and Environmental Sciences, Marche Polytechnic University, Via Brecce Bianche, 60131 Ancona, Italy; 3 Dipartimento di Farmacia, Università di Pisa, Via Bonanno 33, 56126 Pisa, Italy; basma.najar@farm.unipi.it (B.N.); luisa.pistelli@unipi.it (L.P.); 4 National Agricultural Institute of Tunisia, 43 Avenue Charles Nicolle, Tunis 1082, Tunisia; karous-olfa@hotmail.fr; 5 Regional Centre for Research in Horticulture and Organic Agriculture (CRRHAB), Chott Mariem, Sousse 4042, Tunisia; ikbal_c@yahoo.fr

**Keywords:** *Alternaria alternata*, cucurbits, *Cymbopogon citratus*, GC-MS, *Stagonosporopsis cucurbitacearum*

## Abstract

Essential oils represent novel alternatives to application of synthetic fungicides to control against seedborne pathogens. This study investigated seven essential oils for in vitro growth inhibition of the main seedborne pathogens of cucurbits. *Cymbopogon citratus* essential oil completely inhibited mycelial growth of *Stagonosporopsis cucurbitacearum* and *Alternaria alternata* at 0.6 and 0.9 mg/mL, respectively. At 1 mg/mL, *Lavandula dentata*, *Lavandula hybrida*, *Melaleuca alternifolia*, *Laurus nobilis,* and two *Origanum majorana* essential oils inhibited mycelia growth of *A. alternata* by 54%, 71%, 68%, 36%, 90%, and 74%, respectively. *S. cucurbitacearum* mycelia growth was more sensitive to *Lavandula* essential oils, with inhibition of ~74% at 1 mg/mL. To determine the main compounds in these essential oils that might be responsible for this antifungal activity, they were analyzed by gas chromatography–mass spectrometry (GC-MS). *C. citratus* essential oil showed cirtal as its main constituent, while *L. dentata* and *L. nobilis* essential oils showed eucalyptol. The *M. alternifolia* and two *O. majorana* essential oils had terpinen-4-ol as the major constituent, while for *L. hybrida* essential oil, this was linalool. Thus, in vitro, these essential oils can inhibit the main seedborne fungi of cucurbits, with future in vivo studies now needed to confirm these activities.

## 1. Introduction

Cucurbits are an important source of income for countries in the Mediterranean basin, with a total production of nearly 3,356,669 tonnes in 2018 [1]. Squash (*Cucurbita maxima* Duchesne; *Cucurbita moschata* Duchesne) is one of the major cucurbits grown in tropical and temperate regions. *Cucurbita* spp. can be affected by a number of fungal pathogens, which can cause major economic losses [2]. The majority of these fungi are seedborne, such as gummy stem blight (with foliar symptoms) and black rot (with fruit symptoms), which are caused by *Stagonosporopsis cucurbitacearum* (Fr.) Aveskamp, Gruyter & Verkley (anamorph: *Phoma cucurbitacearum* (Fr.) Sacc.), synonym *Didymella bryoniae* (Fuckel) Rehm, and which represent serious diseases that are a major constraint to cucurbit production worldwide [2,3,4]. Nuangmek et al. [5] reported that losses in cantaloupe can also reach 100% under conditions conducive to *S. cucurbitacearum*. *Alternaria alternata* (Fr.) Keissl. is the agent of leaf spot, which is a further major factor responsible for low cucurbit production. The genus *Alternaria* affects the plant seedlings, leaves, stalks, stems, flowers and fruit. Yield losses of 50% and more can occur under weather conditions that are conducive to leaf spot, and in particular temperatures of 25 to 32 °C associated with 40% relative humidity during the day and 95% at night [6]. Many other pathogens have been detected on cucurbits seeds, such as *Fusarium solani* [7,8], *Alternaria cucumerina* [9], *Paramyrothecium roridum*, and *Albifimbria verrucaria* [10,11].

The most important unit of the squash crop is the seed, which should be of high quality and pathogen free. The propagation of such seedborne fungi is generally controlled by chemical treatments [7,12,13]. Indeed, there have been many studies on chemical control against seedborne *S. cucurbitacearum* [14,15,16]. Sudisha et al. [17] reported that seed treatment with the Dithane M-45 (Mancozeb 75% WP) fungicide can reduce the incidence of gummy stem blight in muskmelon crops, although this active ingredient was recently refused approval for use in the European Union, so its use will be banned in few months. Chemical fungicides are generally adopted for disinfestation, disinfection, and protection of seeds and the emerging plantlets. However, these chemicals can also cause environmental pollution due to their high persistence in the soil and water, because of their slow biodegradability [18,19]. 

In recent years, alternatives to synthetic fungicides have been investigated due to the extensive use of fungicides for plant and seed treatments, the problems of pathogen resistance to fungicides that this causes, and the increased demand for organic and free-of-residue vegetables [20,21,22,23]. Natural organic compound, such as plant extracts and essential oils, are among the environmentally friendly alternatives that are being developed and tested for antifungal activities against seedborne pathogens [24]. Essential oils are a rich source of broad-spectrum antifungal plant-derived metabolites that inhibit both fungal growth and their production of toxic metabolites [25]. Tea tree essential oil contains terpinen-4-ol, 1,8-cineole, and γ-terpinene, and at 2%, it has shown potent inhibition of mycelial growth of *Fusarium graminearum, Fusarium culmorum*, and *Pyrenophora graminea* [26]. Riccioni and Orzali [27] reported that tea tree essential oil represents a source of sustainable eco-friendly botanical fungicides, because of its efficacy in the control of seedborne fungi. The genus *Cymbopogon* (Poaceae) is known for its essential oils, especially for extracts of lemongrass (*Cymbopogon citratus* (DC.) Stapf). The in vitro evaluation of the effectiveness of this essential oil on the main seedborne pathogens of cucurbits was reported previously [28,29,30].

The objectives of the present study were to evaluate the inhibitory effects of seven essential oils that differ in their chemical compositions and to determine what the most important compounds in these seven essential oils might be, using gas chromatography–mass spectrometry (GC-MS) analysis.

## 2. Results

### 2.1. In Vitro Inhibition of Fungal Growth by the Seven Essential Oils 

The effects of increasing concentrations of seven essential oils on mycelial growth of the fungi *A. alternata* and *S. cucurbitacearum* were investigated. These essential oils were from various sources, and are defined as (see Table 1): *C.cit*, *Cymbopogon citratus* (lemon grass); *L.dent*, *Lavandula dentata* (lavender); *L.hyb*, *Lavandula hybrida* (lavandin); *M.alt*, *Melaleuca alternifoglia* (tea tree); *L.nob*, *Laurus nobilis* (bay laurel); *O.maj1/2*, *Origanum majorana* 1/2 (majoram). 

**Figure 1:**
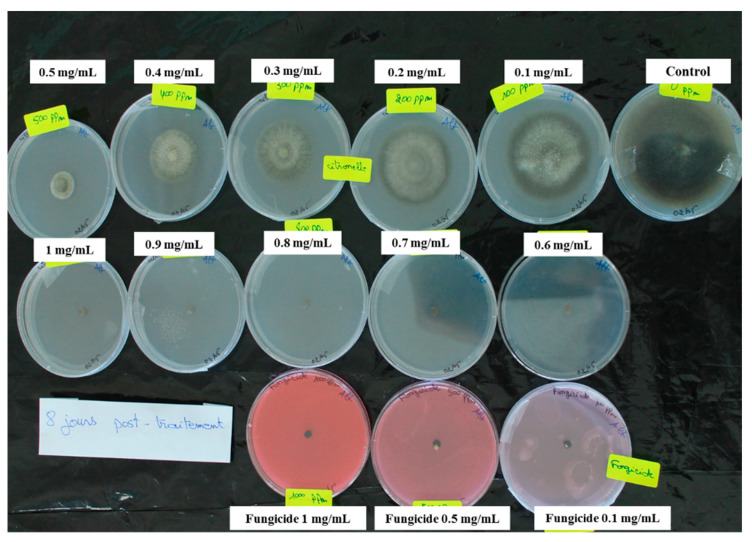
Representative experiment showing inhibition of *Alternaria alternata* mycelial growth by *Cymbopogon citratus* essential oil at 0.1 to 1 mg/mL and by the fungicide combination of 25 g/L difenoconazole plus 25 g/L fludioxonil at 0.1, 0.5 and 1 mg/mL, as seen after 8 days of incubation at 22 ± 2 °C.

**Figure 2:**
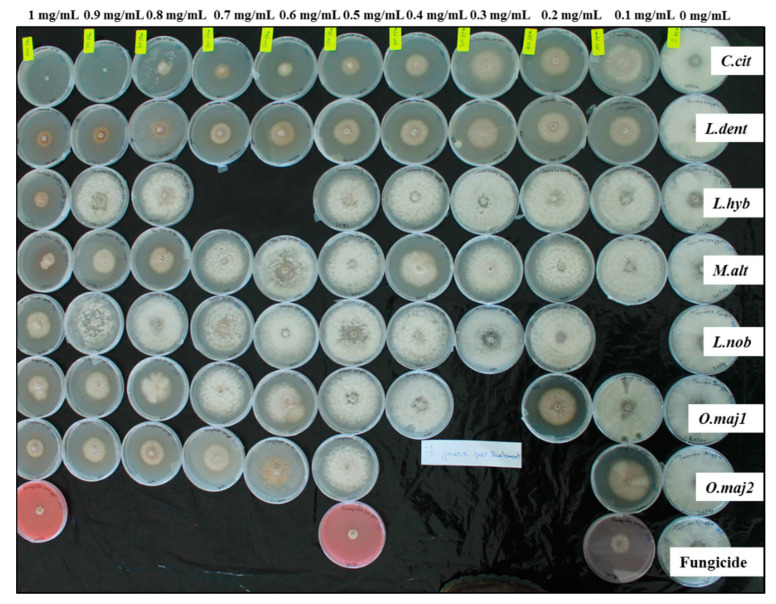
Representative experiment showing inhibition of *Stagonosporopsis cucurbitacearum* mycelial growth by the seven essential oils: *C.cit*, *Cymbopogon citratus*; *L.dent*, *Lavandula dentata*; *L.hyb*, *Lavandula hybrida*; *M.alt*, *Melaleuca alternifolia*; *L.nob*, *Laurus nobilis*; *O.maj1/2*, *Origanum majorana* 1/2, at increasing concentrations (right to left; as indicated) from 0 mg/mL (control) to 1 mg /mL, and by the fungicide combination of 25 g/L difenoconazole plus 25 g/L fludioxonil (positive control) at 0.1, 0.5 and 1 mg/mL, after 7 days of incubation at 22 ± 2 °C.

As can be seen in Figure 1 and Figure 2, and as summarized in Table 2 and Table 3, all of these essential oils inhibited the growth of these two fungi in a dose-dependent manner. The greatest inhibitory activity was shown by the *C.cit* essential oil, with 100% inhibition of mycelial growth of both *A. alternata* and *S. cucurbitacearum* reached at 0.6 mg/mL and 0.9 mg/mL, respectively (Table 2). *A. alternata* was generally more sensitive to these essential oils than *S. cucurbitacearum*, and at 1 mg/mL essential oils, its mycelia growth was inhibited by 55.0%, 71.5%, 68.2%, 36.1%, 74.2%, and 90.5% by *L.dent*, *L.hyb*, *M.alt*, *L.nob*, *O.maj1*, and *O.maj2*, respectively (Table 2). At the same essential oil concentration, *S. cucurbitacearum* radial growth was inhibited by 73.5%, 74.0%, 73.7%, 65.3%, 60.0%, and 67.3%, respectively (Table 3). The positive control of the fungicide combination of 25 g/L difenoconazole plus 25 g/L fludioxonil completely inhibited the mycelial growth of *A. alternata* at all concentrations tested. Against *S. cucurbitacearum*, this fungicide combination at 0.1, 0.5, and 1 mg/mL inhibited the mycelial growth by 75.7%, 84.9%, and 86.7%, respectively. 

**Figure 3:**
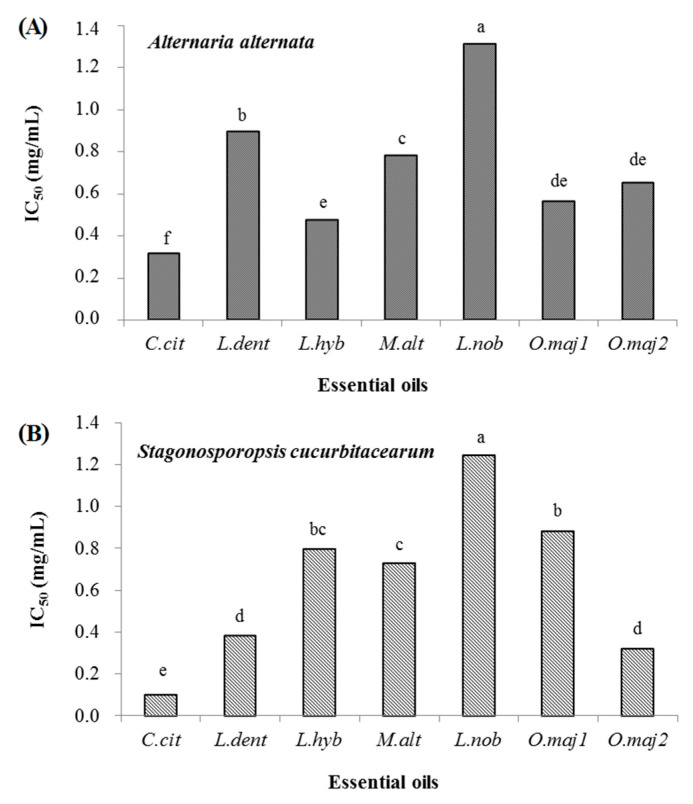
Inhibitory concentration for 50% reduction (IC_50_) of mycelial growth of *Alternaria alternata* (**A**) and *Stagonosporopsis cucurbitacearum* (**B**) by the seven essential oils: *C.cit, Cymbopogon citratus; L.dent*, *Lavandula dentata; L.hyb*, *Lanvandula hybrida; M.alt*, *Melaleuca alternifolia*; *L.nob*, *Laurus nobilis; and O.maj1/2, Origanum majorana* 1/2. Data with different letters (**a**–**f**) are significantly different between treatments (*p* ≤ 0.05; Fisher’s LSD).

In addition, the *C.cit* essential oil had a fungicidal effect against *S. cucurbitacearum* from 900 µg/mL. Indeed, for *A. alternata*, *C.cit* had fungistatic effects at 0.6 mg/mL and 0.7 mg/mL, and it was fungicidal from 0.8 mg/mL (Table 4). These data show that the *C.cit* had potent antifungal activity against *A. alternata* and *S. cucurbitacearum* with IC_50_ values of 0.315 mg/mL and 0.102 mg/mL, respectively (Figure 3). The essential oils of *L.dent*, *L.hyb*, *M.alt*, *O.maj1*, and *O.maj2* showed moderate antifungal activities against *A. alternata*, with IC_50_ values from 0.473 mg/mL to 0.893 mg/mL, as similarly against *S. cucurbitacearum*, with IC_50_ values from 0.322 mg/mL to 0.884 mg/mL. However, *L.nob* showed only weak antifungal activities against both *A. alternata* and *S. cucurbitacearum*, as seen by its relatively high IC_50_ values of 1.310 mg/mL and 1.248 mg/mL, respectively (Figure 3). 

### 2.2. Chemical Profiles of the Essential Oils

The 41 components given in Table 5 were identified as comprising from 97.7% (*O.maj1*) to 100% (*L.hyb*) of these essential oils. The oxygenated monoterpenes dominated in all of the essential oils, even though these belonged to different plant families and species. They represented more than two-thirds of the fraction in three of the four *Lamiaceae*: *L.dent* (81.1%), *L.hyb* (90.8%), and *O.maj2* (66.8%). The oxygenated monoterpenes were also the highest proportionally in *C.cit* (88.5%) and *L.nob* (70.3%). On the other hand, the compositions of *M.alt* and *O.maj1* were divided mainly between oxygenated monoterpenes as the main class (48.1%, 49.7%, respectively) and monoterpene hydrocarbons in similar proportions (40.4%, 44.3%, respectively). In more detail, *C.cit* showed α-citral (geranial; 51.6%) and β-citral (neral; 26.0%), whereby these two major oxygenated monoterpenes represented together over three-quarters of the total composition. In the *Lamiaceae*, almost two-thirds of *L.dent* was eucalyptol (63.5%) and β-selinene (4.1%). Instead, the total composition of *O.maj1* and *O.mag2* included around half and over two-thirds as terpenen-4-ol (44.8%) and *p*-cymene (68.2%), respectively, followed by γ-terpinene (12.6%) for *O.maj1* and α-terpineol (5.4%) for *O.maj2*. For the two commercial essential oils, the main compounds of *L.hyb* were linalool (33.7%) and linalyl acetate (27.7%), followed by camphor (9.3%), while *M.alt* showed terpinen-4-ol (41.1%) as 86% of its oxygenated monoterpene, with γ-terpinene (16.0%), p-cymene (9.3%), and α-terpinene (6.1%), together representing 78% of the monoterpene hydrocarbons. Finally, more than half of the identified fractions of the *Lauraceae L.nob* were characterized by the combination of eucalyptol (47.9%) and α-terpinyl acetate (10.2%).

## 3. Discussion

In this study, these in vitro assays for the antifungal activities of these seven essential oils on mycelial growth of two fungi showed that the lemongrass (*C.cit*) essential oil was the most effective. The mycelial growth of *A. alternata* was totally inhibited by application of *C.cit* at a moderate concentration, while *S. cucurbitacearum* was completely inhibited at the highest *C.cit* concentration, with fungicidal activity seen in both cases. Only a few studies have investigated lemongrass essential oils and these fungi, with most studies focused on the essential oil activity rather than its composition. Shafique et al. [29] reported total inhibition of *A. alternata* by a *C. citratus* essential oil, with an IC_50_ of 279.13 µL/L, as also reported by Jie et al. [31]. In the same year, Guimarães et al. [32] confirmed in vitro fungitoxic activity on *A. alternata*, with the essential oil rich in citral (69.3%) and myrcene (23.8%). For the second fungus here, *S. cucurbitacearum*, Fiori et al. [30] reported 100% inhibition of mycelial growth and spore germination at a rate of 20 µL *C. citratus* essential oil. Seixas et al. [28] reported the same result at 0.25, 0.5, 0.75, 1, and 1.25 mg/mL *C. citratus* essential oil. A number of studies have reported these high proportions of the two isomers α-citral and β-citral in *C. citratus* essential oils, even when collected from different countries [33,34,35,36,37], as also confirmed by the present study. Brügger et al. [38] reported that in addition to the high proportion of citral, their commercial *C. citratus* essential oil showed relevant amounts of nonan-4-ol (6.5%) and camphene (5.2%). These two compounds were completely absent in the *C.cit* essential oil used in the present study. A Brazilian commercial *C. citratus* essential oil also indicated a different composition, which was rich in nonterpenes, as especially 4,8-dimethyl-3,7-nonadien-2-one (25.0%), 1-heptadec-1-ynyl-cyclopentanol (9.6%), and 7,7-dimethyl-bicycloheptan-2-ol (8.0%); here, the proportion of citral was less than 37% [39]. The antifungal activity of *C. citratus* essential oil has also been reported against other fungi, including *Aspergillus flavus*. This activity can be ascribed to the presence of various components such as citral, geraniol, and β-myrcene [37,40]. According to some studies, citral and geranol can indeed inhibit the mycelial growth of *Fusarium oxysporum*, *Colletotrichum gloeosporioides*, *Bipolaris* sp. and *A. alternata* [41,42]. These major compounds in the *C. citratus* essential oil also have antioxidant and antimicrobial activities [43]. Furthermore, Kurita et al. [44] defined the fungicidal action of citral as due to its ability to receive electrons from the fungus cell, through charge transfer with an electron donor in the cell, which results in death of the fungus. In previous studies, β-myrcene and geraniol were found in *C.cit* essential oil. These compounds with citral can contributed to inhibit the mycelial growth of *A. alternata* and *S. cucurbitacearum*.

The cultivated lavender *L. dentata* essential oil showed eucalyptol (63.5%) as the major compound in the present study, which was higher than that previously reported for both inflorescences (46.3%) and the aerial parts (40.4%) [45]. Iranian lavandin (*L. hybrida*) has also been characterized by high proportions of oxygenated monoterpenes, with eucalyptol (41.1%) as the main component, followed by borneol (20.7%) and camphor (10.8%) [46]. All of these components were present in *L.hyb* in the present study, although in lower amounts (6.5%, 4.4%, 9.3%, respectively), with the main component here being linalool (33.7%) and linalyl acetate (27.7%). The antifungal activity of linalool on *Candida* species was recently studied by Dias et al. [47], who indicated the potential use of this unsaturated monoterpene as a strong candidate with antifungal potency. According to Pitarokili et al. [48], linalyl acetate was inactive against all of the fungi they studied, although it showed weak activity against only *Sclerotinia sclerotiorum*. On the contrary, they confirmed the antifungal effects of linalool.

Good antifungal effects on mycelial growth of *A. alternata* were also seen here using the *Origanum* essential oils. Even though the *O. majorana* essential oils tested here had the same classes of compounds shown in a Brazilian species studied by Chaves et al. [49], they did not show pulegone as the main compound. An Italian species investigated by Della Pepa et al. [50] was in partial agreement with the present study for the high amount of terpinen-4-ol (29.6%), while a Tunisian oregano species were characterized by similar terpinen-4-ol content [51,52]. Moreover, Busatta et al. [53] showed that an Egyptian essential oil that was extracted by hydrodistillation of dried leaves of *O. majorana* showed the same dominance of terpinen-4-ol (31.8%) and γ-terpinene (13.0%). Although most studies on the composition of *Origanum* essential oils have agreed on the main compounds from *O. majorana* [54,55,56,57], these have indeed varied. The effectiveness of *Origanum* might be due to its high content of terpinen-4-ol, a monoterpene alcohol that is known to have good antifungal activity, as previously reported against *Fusarium avenaceum*, *Fusarium moniliforme*, *Fusarium semitectum*, *F. solani*, *F. oxysporum*, and *F. graminearum* [58,59]. Its fungicidal activity was also reported by Morcia et al. [60], who analyzed its potency on mycotoxigenic plant pathogens. However, Ebani et al. [61] showed weak activity of a tea tree essential oil on *Aspergillus fumigatus* even though terpinen-4-ol was present at relatively high levels. This might be explained by the synergistic effects among all of the different components in each of the essential oils. 

The *M. alternifolia* essential oil in the present study was characterized by high levels of terpinen-4-ol (41.1%) and γ-terpinene (16.0%). These are comparable with the data reported by Elmi et al. [62] and Silva et al. [63]. In their investigations of Italian and Brazilian, commercial essential oils, they confirmed the predominance of terpinen-4-ol (41.5%, 43.1%, respectively) and γ-terpinene (20.6%, 22.8%, respectively). They also reported relatively high levels of both α-terpinene (9.6%, 9.3%, respectively) and α-terpineol (4.4%, 5.2%, respectively), while in the present study these two compounds were present in lower amounts (6.1%, 3.7%, respectively). It is also interesting to note the high proportion of p-cymene (9.3%) in the present study. In a more recent study, α-terpineol (4.4%) and 1,8-cineol (4.0%) were found in relatively high amounts, together with terpinen-4-ol (30.2%) and γ-terpinene (16.9%) [61]. In the present study, 1,8-cineol was also present but at a lower amount (2.8%). 

The *L.nob* profile in the present study was in good agreement with that reported by Dhifi et al. [64], where they also showed high proportions of oxygenated monoterpenes (64.3%), with eucalyptol as the main constituent (46.8%) in their Tunisian species. 

## 4. Materials and Methods 

### 4.1. Origin of the Essential Oils 

The lemongrass (*Cymbopogon citratus* (DC.) Stapf), lavender (*Lavandula dentata* L.), sweet marjoram (*Origanum majorana* L.), and bay laurel (*Laurus nobilis* L.) essential oils were provided by different laboratories (see Table 1), where the dried aerial parts of the plants were hydrodistilled using a Clevenger apparatus, as recommended by the European Pharmacopeia. The lavandin (*Lavandula hybrida* E.Rev. ex Briq) and tea tree (*Melaleuca alternifolia* (Maiden & Betche) Cheel) essential oils were from Flora Srl (Lorenzana, Pisa, Italy). The selection of these essential oils was based initially on their availability in our laboratory, and then on the studies in the literature that have reported in vitro activities of some of these against pathogen growth [65,66,67].

### 4.2. Fungal Strains

The *A. alternata* (GenBank accession: MK497774) and *S. cucurbitacearum* (GenBank accession: MF401569) strains used in the present study were isolated from infected squash seeds [2]. Pure cultures were transferred into Petri dishes (diameter, 90 mm) with potato dextrose agar (PDA; 42 g/L; Liofilchem Srl, Roseto degli Abruzzi, Italy) and incubated at 22 ± 2 °C with a photoperiod of 12/12 h dark/ ultraviolet light (TL-D 36W BLB 1SL; Philips, Dublin, Ireland).

### 4.3. In Vitro Antifungal Activities on Mycelial Growth

The antifungal activities of these *C.cit*, *L.dent*, *L.hyb*, *O.maj1, O.maj2*, *M.alt*, and *L.nob* essential oils were determined according to their contact phase effects on mycelial growth of *A. alternata* and *S. cucurbitacearum*. For these tests, the essential oils were dissolved in sterilized distilled water with 0.1% (*v*/*v*) Tween 20 (Sigma Aldrich, Steinheim, Germany), to obtain homogeneous emulsions. The autoclaved PDA medium (cooled to 40 °C) had the essential oil emulsions added to obtain the final concentrations of 0.1, 0.2, 0.3, 0.4, 0.5, 0.6, 0.7, 0.8, 0.9, and 1 mg/mL. The negative control was PDA containing 0.1% (*v*/*v*) Tween 20. The positive control was provided by three concentrations (0.1, 0.5, 1 mg/mL) of fungicides as 25 g/L difenoconazole plus 25 g/L fludioxonil (Celest Extra 50 FS; Cambridge, UK). The PDA was mixed and poured immediately into Petri dishes (diameter, 90 mm; 20 mL/plate), and after medium solidification, each plate was inoculated under aseptic conditions with 6 mm plugs of *A. alternata* or *S. cucurbitacearum*, taken from the edges of actively growing cultures. The experiments were carried out as three replicates per concentration and treatment. The inoculated plates were sealed with Parafilm and incubated for 7 days at 22 ± 2 °C with a photoperiod of 12/12 h dark/ ultraviolet light (TL-D 36W BLB 1SL; Philips, Dublin, Ireland). The orthogonal diameters of the colonies were measured daily until the control plates were completely covered by the mycelia. Mycelial growth inhibition was calculated based on Equation (1):

Mycelial growth inhibition (%) = [(dc − dt)/dc] × 100(1)

where dc and dt represent the mean diameter of the mycelial growth of the control and treated fungal strains, respectively. Moreover, the IC_50_ for mycelial growth inhibition of the fungi was determined from the linear regression between the essential oil concentrations and the mycelial growth inhibition.

Experiments were performed to differentiate between the fungicidal and fungistatic activities of the elevated essential oil concentrations against fungi. Here, each of the completely inhibited fungal plugs were transferred to fresh PDA plates to note their viability after 7 days of incubation under the same conditions. 

### 4.4. Gas Chromatography-Mass Spectrometry Analysis

The volatile constituents of each essential oil were analyzed by GC-MS as previously reported [68]. They were processed using a gas chromatograph (Agilent 7890B; Agilent Technologies Inc., Santa Clara, CA, USA) equipped with a capillary column (Agilent HP-5MS; 30 m × 0.25 mm; coating thickness, 0.25 μm; Agilent Technologies Inc., Santa Clara, CA, USA) and a single quadrupole mass detector (Agilent 5977B; Agilent Technologies Inc., Santa Clara, CA, USA). The analytical conditions were as follows: injector temperature, 220 °C; transfer line temperature, 240 °C; oven temperature programmed, from 60 °C to 240 °C at 3 °C/min; carrier gas, helium at 1 mL/min; injection volume, 1 μL (in 0.5% HPLC grade n-hexane solution); split ratio, 1:25. The full scan acquisition parameters were as follows: scan range, 30 *m/z* to 300 *m/z*; scan time, 1.0 s (See supplementary materials). 

Identification of the constituents was based on comparisons of retention times with those of the authentic standards, with comparisons of their linear retention indices relative to the series of n-hydrocarbons. Computer matching was also used against commercial (NIST 14, Adams) and laboratory developed mass spectra libraries built for pure substances and components of known oils, and against the mass spectrometry literature data [69,70,71,72,73,74].

### 4.5. Statistical Analysis

Analysis of variance was calculated using SPSS (version 20, IBM, Armonk, NY, USA). The data were analyzed by analysis of variance (ANOVA). Means were compared using Fisher’s test protected least significant difference at *p* ≤ 0.05. All of the trials were repeated at least twice, and data are means ± standard error (SE).

## 5. Conclusions

The management of plant diseases using natural compounds is a great and important need nowadays. The present study has demonstrated the in vitro activities of seven essential oils and their efficacies against the fungi *A. alternata* and *S. cucurbitacearum*. These data show that the chemical compositions of essential oils can affect their antimicrobial activities. These essential oils were characterized by high proportions of oxygenated monoterpenes followed by monoterpene hydrocarbons. Essential oil with citral, β-myrcene, and geraniol as major components (i.e., lemongrass [*C.cit*],) controlled these fungi most effectively, followed by essential oils containing terpinen-4-ol or linalool (i.e., marjoram [*O.maj1/2*], tea tree [*M.alt*], lavandin [*L.hyb*]). Antifungal activity of essential oils can be ascribed to individual effect of major components, and/or due to a synergistic effect of its minor components. Further studies are required to determine the effects of these oils as seed treatments, to evaluate their potential as preventive and curative treatments.

## Figures and Tables

**Table 1:** Details of the essential oils included in this study.

Code	Species	Family	Common Name	Source
***C.cit***	*Cymbopogon citratus* (DC.) Stapf	*Poaceae*	Lemongrass	Biopesticides Laboratory, Regional Centre for Research in Horticulture and Organic Agriculture (CRRHAB), Tunisia
***L.dent***	*Lavandula dentata* L.	*Lamiaceae*	Lavender	CRRHAB, Tunisia
***L.hyb***	*Lavandula hybrida* E.Rev. ex Briq	*Lamiaceae*	Lavandin	FLORA s.r.l. (Batch N° 161808)
***M.alt***	*Melaleuca alternifolia* (Maiden & Betche) Cheel	*Myrtaceae*	Tea tree	FLORA s.r.l. (Batch N° 161960)
***L.nob***	*Laurus nobilis* L.	*Lauraceae*	Bay laurel	Medicinal Plants Laboratory, National Institute of Agronomy of Tunisia (INAT)
***O.maj1***	*Origanum majorana* L.	*Lamiaceae*	Marjoram	INAT
***O.maj2***	*Origanum majorana* L.	*Lamiaceae*	Marjoram	CRRHAB, Tunisia

**Table 2:** Mycelial growth inhibition of *Alternaria alternata* by the seven essential oils. *C.cit*, *Cymbopogon citratus*; *L.dent*, *Lavandula dentata*; *L.hyb*, *Lanvandula hybrid*; *M.alt*, *Melaleuca alternifolia*; *L.nob*, *Laurus nobilis*; *O.maj1/O.maj2*, *Origanum majorana* 1/2, after 7 days of incubation at 22 ± 2 °C.

Essential Oil	Inhibition of Mycelial Growth of *Alternaria alternata* (%) at Increasing Essential Oil Concentrations (mg/mL)										
	0	0.1	0.2	0.3	0.4	0.5	0.6	0.7	0.8	0.9	1
*C.cit*	0.00	20.28 ± 4.32	25.03 ± 1.56	43.21 ± 2.33	65.92 ± 6.29	79.14 ± 10.97	100	100	100	100	100
*L.dent*	0.00	9.95 ± 2.30	20.07 ± 3.63	29.99 ± 4.97	44.44 ± 3.13	43.82 ± 1.84	43.00 ± 3.18	37.63 ± 1.65	44.44 ± 0.21	43.00 ± 1.89	54.98 ± 0.83
*L.hyb*	0.00	31.23 ± 4.40	32.88 ± 4.72	42.38 ± 2.18	50.64 ± 2.89	51.05 ± 2.50	59.31 ± 3.76	63.44 ± 1.64	65.72 ± 1.49	67.37 ± 0.55	71.50 ± 0.95
*M.alt*	0.00	13.47 ± 5.36	15.74 ± 3.06	21.52 ± 10.17	21.73 ± 5.38	24.00 ± 1.09	25.44 ± 4.42	29.37 ± 3.45	62.00 ± 1.49	67.99 ± 2.33	68.19 ± 2.98
*L.nob*	0.00	0.58 ± 0.29	0.58 ± 0.29	1.28 ± 0.41	8.72 ± 2.51	12.02 ± 1.56	23.17 ± 2.48	24.00 ± 1.65	25.03 ± 3.41	33.91 ± 4.96	36.08 ± 2.44
*O.maj1*	0.00	20.07 ± 2.18	37.84 ± 9.68	42.59 ± 2.30	45.48 ± 4.13	47.13 ± 1.80	47.75 ± 1.09	53.12 ± 7.10	60.97 ± 3.41	64.27 ± 3.25	74.18 ± 1.45
*O.maj2*	0.00	27.10 ± 0.55	31.85 ± 1.99	32.47 ± 1.99	33.91 ± 0.55	37.01 ± 0.41	42.38 ± 1.24	42.59 ± 0.83	46.92 ± 0.55	63.86 ± 1.61	90.50 ± 9.50
Fungicides ^a^	0.00	100	-	-	-	100	-	-	-	-	100

Data are means ±SD (*n* = 3). ^a^ 25 g/L difenoconazole + 25 g/L fludioxonil. - not tested.

**Table 3:** Mycelial growth inhibition of *Stagonosporopsis cucurbitacearum* by the seven essential oils. *C.cit*, *Cymbopogon citratus*; *L.dent*, *Lavandula dentata*; *L.hyb*, *Lanvandula hybrid*; *M.alt*, *Melaleuca alternifolia*; *L.nob*, *Laurus nobilis*; *O.maj1/O.maj2*, *Origanum majorana* 1/2, after 7 days of incubation at 22 ± 2 °C.

Essential Oil	Inhibition of Mycelial Growth of *Stagonosporopsis cucurbitacearum* (%) at Increasing Essential Oil Concentrations (mg/mL)										
	0	0.1	0.2	0.3	0.4	0.5	0.6	0.7	0.8	0.9	1
*C.cit*	0.00	51.76 ± 3.86	53.53 ± 1.22	58.24 ± 0.59	67.25 ± 1.37	74.51 ± 3.08	85.49 ± 7.45	82.16 ± 9.00	87.06 ± 6.51	100	100
*L.dent*	0.00	29.41 ± 0.00	41.18 ± 6.79	47.06 ± 1.36	51.76 ± 0.68	61.37 ± 0.71	62.75 ± 0.71	66.27 ± 0.39	71.37 ± 0.78	73.33 ± 0.39	73.53 ± 0.59
*L.hyb*	0.00	5.49 ± 1.68	6.27 ± 2.18	10.39 ± 2.05	12.75 ± 1.04	21.57 ± 5.85	22.75 ± 4.43	27.65 ± 4.75	53.92 ± 0.52	63.73 ± 0.85	73.92 ± 3.63
*M.alt*	0.00	3.92 ± 1.96	22.16 ± 1.04	20.78 ± 3.08	20.59 ± 3.02	30.78 ± 3.16	31.37 ± 4.11	41.96 ± 3.35	60.20 ± 0.71	63.92 ± 0.20	73.73 ± 0.85
*L.nob*	0.00	0.98 ± 0.71	3.73 ± 3.73	5.88 ± 2.38	5.88 ± 2.37	5.69 ± 0.52	6.67 ± 0.71	11.37 ± 4.37	15.29 ± 4.57	29.22 ± 8.35	65.29 ± 3.83
*O.maj1*	0.00	0.00	5.69 ± 1.87	8.24 ± 1.22	9.02 ± 1.19	10.98 ± 1.37	33.73 ± 4.31	33.73 ± 3.74	47.45 ± 6.97	54.12 ± 2.96	60.00 ± 1.56
*O.maj2*	0.00	34.12 ± 4.42	49.22 ± 1.41	54.31 ± 4.27	56.47 ± 1.96	57.25 ± 1.56	58.82 ± 1.04	59.61 ± 1.80	61.57 ± 1.19	63.53 ± 0.34	67.25 ± 1.74
Fungicides ^a^	0.00	75.69 ± 0.00	-	-	-	84.90 ± 0.68	-	-	-	-	86.67 ± 0.39

Data are means ±SD (*n* = 3). ^a^ 25 g/L difenoconazole + 25 g/L fludioxonil. - not tested.

**Table 4:** Fungistatic and fungicidal activities of *Cymbopogon citratus* essential oil on mycelia growth of *Stagonosporopsis cucurbitacearum* and *Alternaria alternata* after 7 days of incubation at 22 ± 2 °C.

Fungus	Fungicidal and Fungistatic Activities of *Cymbopogon citratus* Essential Oil at Increasing Concentrations (mg/mL)									
	0.6		0.7		0.8		0.9		1	
	Fungicidal	Fungistatic	Fungicidal	Fungistatic	Fungicidal	Fungistatic	Fungicidal	Fungistatic	Fungicidal	Fungistatic
*Alternaria alternata*	NO	Yes	NO	Yes	Yes	NO	Yes	NO	Yes	NO
*Stagonosporopsis cucurbitacearum*	-	-	-	-	-	-	Yes	NO	Yes	NO

- not tested.

**Table 5:** Relative levels of the volatile constituents of the different essential oils determined by gas chromatography–mass spectrometry analysis.

Compound ^a^		Class	Linear Retention Index		*Relative amount (%)*						
			(*n*-alkane) ^b^	(Adams, 2007) ^c^	*Poaceae*	*Lamiaceae*				*Myrtaceae*	*Lauraceae*
					*C.cit*	*L.dent*	*L.hyb*	*O.maj1*	*O.maj2*	*M.alt*	*L.nob*
**1**	α-Pinene	mh ^d^	937	932	0.1	0.6	0.5	0.6	0.3	2.7	5.6
**2**	Sabinene	mh	974	969	0.1	-	0.1	4.5	2.2	-	6.7
**3**	β-Pinene	mh	979	974	-	3.1	0.5	0.4	0.2	0.7	5.0
**4**	β-Myrcene	mh	991	988	5.3	-	0.4	1.1	0.6	0.6	1.2
**5**	α-Terpinene	mh	1017	1014	-	-	-	5.7	0.8	6.1	0.8
**6**	*p*-Cymene	mh	1025	1020	0.3	0.9	0.2	11.3	17.8	9.3	0.6
**7**	Limonene	mh	1030	1024	0.4	1.1	0.8	3.5	2.4	1.0	1.7
**8**	Eucalyptol	om	1032	1026	0.6	63.5	6.5	0.7	0.2	2.8	47.9
**9**	γ-Terpinene	mh	1060	1054	-	-	-	12.6	3.8	16.0	1.4
**10**	Terpinolene	mh	1088	1086	-	-	0.2	3.4	1.2	3.0	0.3
**11**	Linalool	om	1099	1095	0.8	1.8	33.7	1.1	3.6	-	7.4
**12**	*cis*-*p*-Menth-2-en-1-ol	om	1122	1118	-	-	-	1.3	0.7	0.1	-
**13**	*trans*-Pinocarveol	om	1139	1135	-	2.9	-	-	-	-	-
**14**	*trans*-*p*-Menth-2-en-1-ol	om	1141	1136	-	-	-	1.2	0.7	0.1	-
**15**	Camphor	om	1145	1141	-	0.5	9.3	-	0.1	-	-
**16**	γ-Terpineol	om	1166	1162	-	1.3	-	-	-	-	0.3
**17**	*endo*-Borneol	om	1167	1165	-	0.4	4.4	0.1	0.2	-	-
**18**	*p*-Mentha-1,5-dien-8-ol	om	1170	1166	2.5	-	-	-	-	-	-
**19**	Terpinen-4-ol	om	1177	1174	0.1	1.3	4.5	32.4	50.1	41.1	1.5
**20**	*p*-Cymen-8-ol	om	1183	1179	1.1	0.3	-	0.3	0.4	-	-
**21**	Cryptone	nt	1186	1183	-	1.3	-	-	-	-	-
**22**	α-Terpineol	om	1189	1186	-	1.8	1.1	6.0	5.4	3.7	1.6
**23**	Myrtenal	om	1198	1195	-	2.7	-	-	-	-	-
**24**	*trans*-Piperitol	om	1208	1207	-	-	-	1.0	0.6	-	-
**25**	β-Citral	om	1240	1235	26.0	-	-	-	-	-	-
**26**	Carvone	om	1243	1239	0.9	1.6	-	0.2	0.6	-	-
**27**	Geraniol	om	1253	1249	2.7	-	-	-	-	-	-
**28**	Linalyl acetate	om	1257	1254	-	-	27.7	2.7	2.2	-	0.2
**29**	α-Citral	om	1270	1264	51.6	-	-	-	-	-	-
**30**	2-Undecanone	nt	1294	1293	1.2	-	-	-	-	-	-
**31**	4-Terpinyl acetate	om	1300	1300	-	-	-	1.5	1.0	-	-
**32**	Lavandulyl acetate	om	1304	1288	-	-	3.0	-	-	-	-
**33**	𝛼-Terpinyl acetate	om	1350	1346	-	-	-	0.1	-	-	10.2
**34**	Methyleugenol	pp	1404	1403	-	-	-	-	-	-	3.1
**35**	β-Caryophyllene	sh	1419	1417	-	-	2.0	2.4	1.6	0.5	0.5
**36**	Aromandendrene	sh	1440	1439	-	-	-	-	-	2.2	-
**37**	β-Selinene	sh	1486	1489	-	4.1	-	-	-	0.1	-
**38**	Eremophyllene	sh	1498	1492	-	-	-	-	-	1.5	-
**39**	δ-Cadinene	sh	1524	1522	-	-	-	-	-	1.7	0.1
**40**	Caryophyllene oxide	os	1583	1582	0.2	1.9	0.1	0.2	0.3	-	-
**41**	β-Eudesmol	os	1651	1649	-	2.1	-	-	-	-	-
**Monoterpene Hydrocarbons**		mh			6.5	5.9	3.6	44.3	29.7	40.4	24.0
**Oxygenated Monoterpenes**		om			88.5	81.1	90.8	49.7	66.8	48.1	70.3
**Sesquiterpene Hydrocarbons**		sh			-	5.5	4.0	3.3	2.0	9.3	1.2
**Oxygenated Sesquiterpens**		os			0.3	4.6	0.2	0.6	1.1	1.8	-
**Penylpropanoids**		pp			-	-	-	-	-	-	3.6
**Non-terpene Derivatives**		nt			3.1	2.2	1.4	-	-	-	0.4
**Total Identified**					98.4	99.3	100.0	97.7	99.6	99.6	99.5

^a^ Compounds present at ≥1% in at least one of the analyzed essential oils. C.cit: Cympobogon citratus; L.dent: Lavandula dentata; L.hyb: Lavandula hybrida; O.maj1/2: Origanum majorana 1/2; M.alt: Melaleuca alternifolia; L.nob: Laurus nobilis. ^b^ Linear retention index relative to n-alkane on the DB5 column. ^c^ Linear retention index reported by Adams (2007). ^d^ mh: monoterpene hydrocarbons; om: oxygenated monoterpenes; os: oxygenated sesquiterpens; sh: sesquiterpene hydrocarbons; pp: penylpropanoids; nt: nonterpene derivatives.

## Data Availability

Data is contained within the article or supplementary material.

## Supplementary Materials

The following are available online https://www.mdpi.com/2079-6382/10/2/104/s1. Figure S1. Chromatogram of *Cympobogon citratus*. Figure S2. Chromatogram of *Lavandula dentata*. Figure S3. Chromatogram of *Lavandula hybrida*. Figure S4. Chromatogram of *Origanum majorana*1. Figure S5. Chromatogram of *Origanum majorana*2. Figure S6. Chromatogram of *Melaleuca alternifolia*. Figure S7. Chromatogram of *Laurus nobilis*.
