# Supplementary material for: Antifungal Activity and Chemical Composition of Seven Essential Oils to Control the Main Seedborne Fungi of Cucurbits
Source: Antibiotics (Basel). 2021 Jan 22;10(2):104. doi: 10.3390/antibiotics10020104 (PMC7912402; doi:10.3390/antibiotics10020104)
Supplement: Supplementary file 1 [file antibiotics-10-00104-s001.pdf]

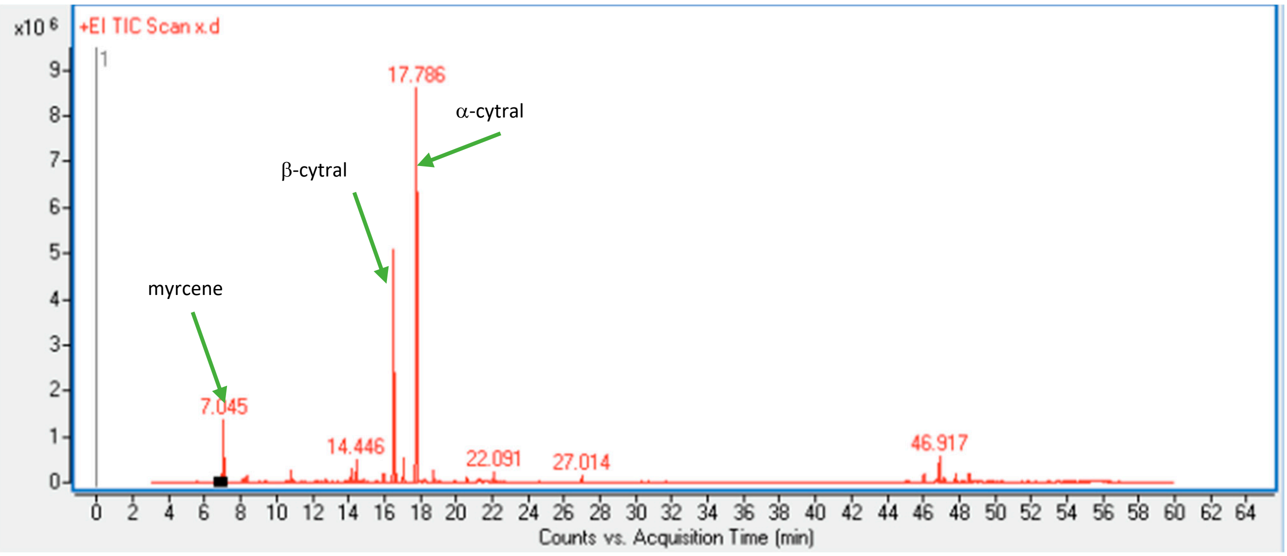

Figure S1. Chromatogram of *Cympobogon citratus* EO

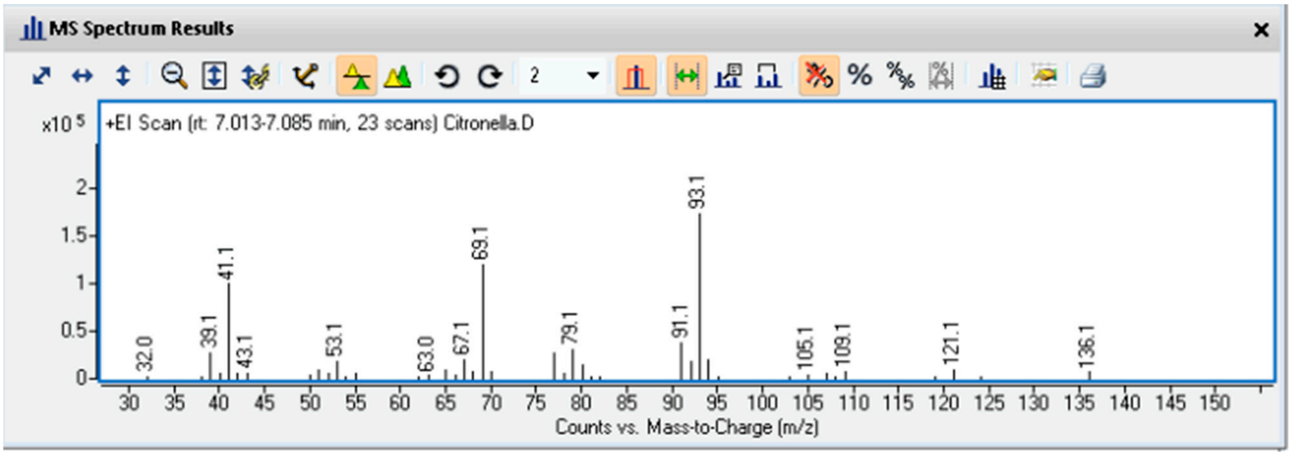

Figure S1. a. MS spectrum of myrcene

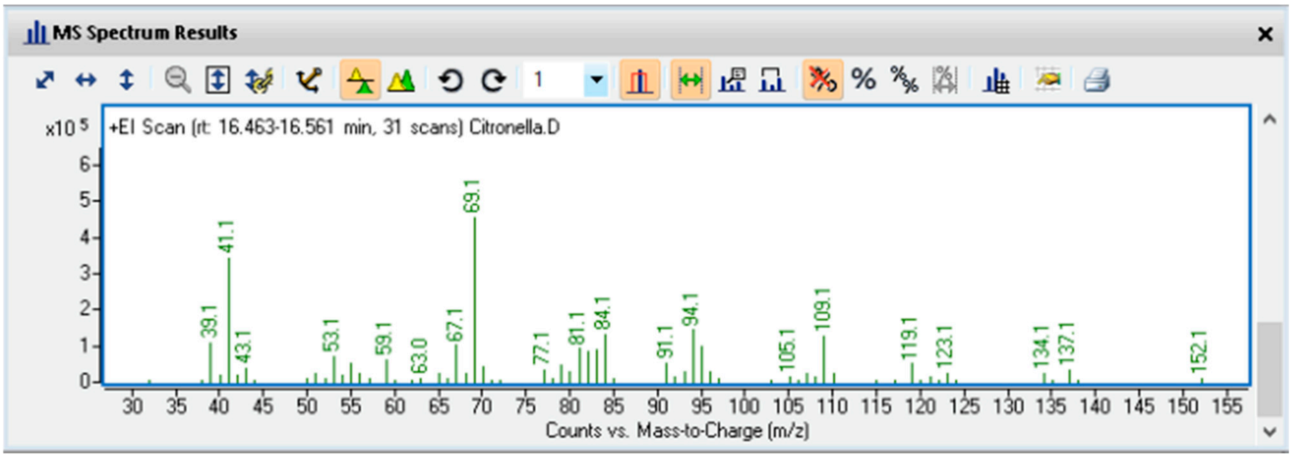

Figure S1. b. MS spectrum of b-citral

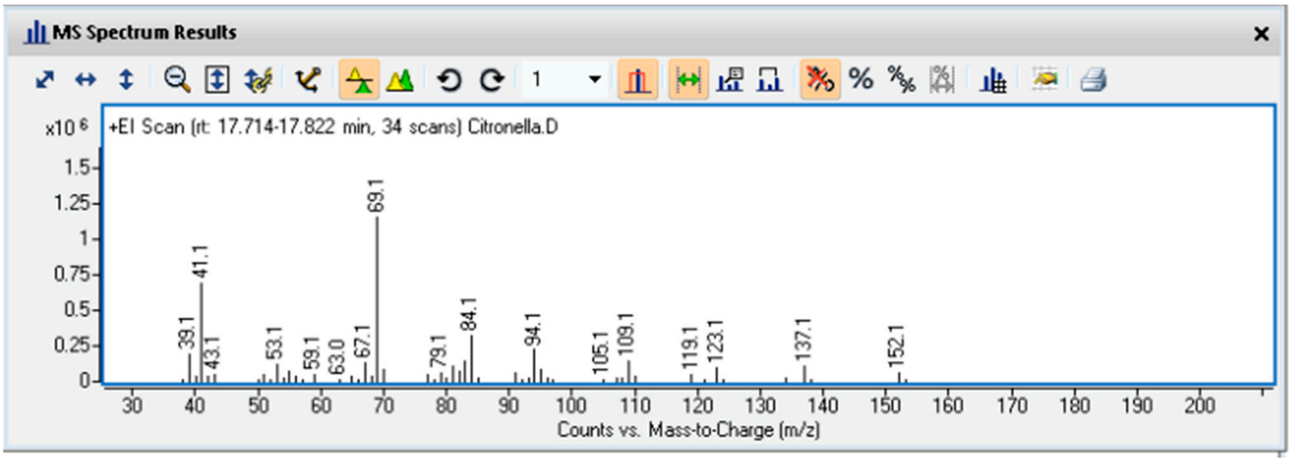

Figure S1. c. MS spectrum of a-citral

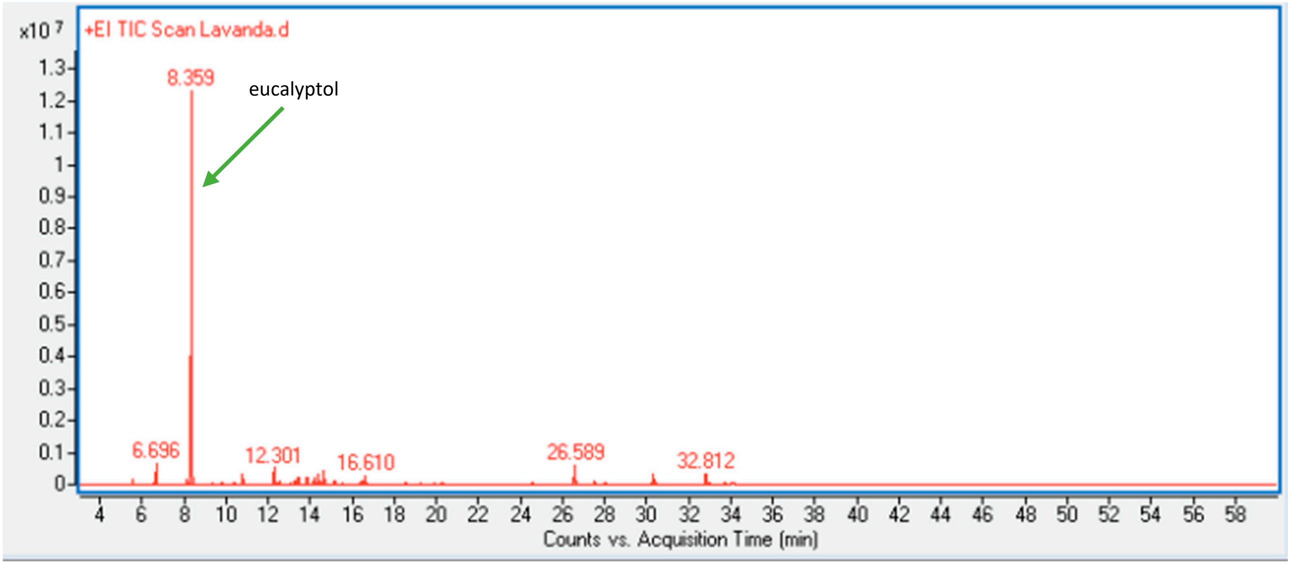

Figure S2. Chromatogram of *Lavandula dentata* EO

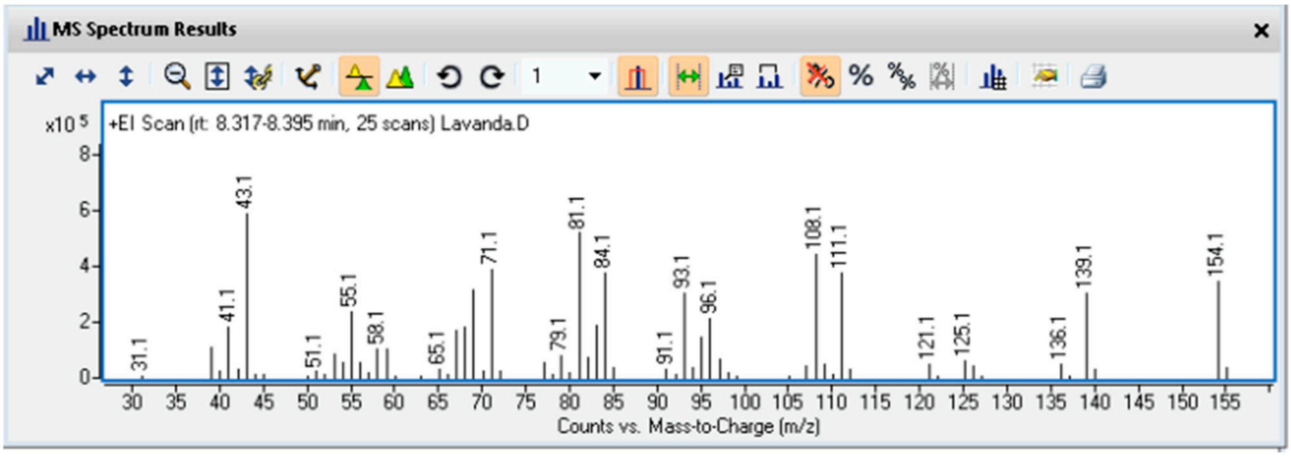

Figure S2. a. MS spectrum of eucalyptol

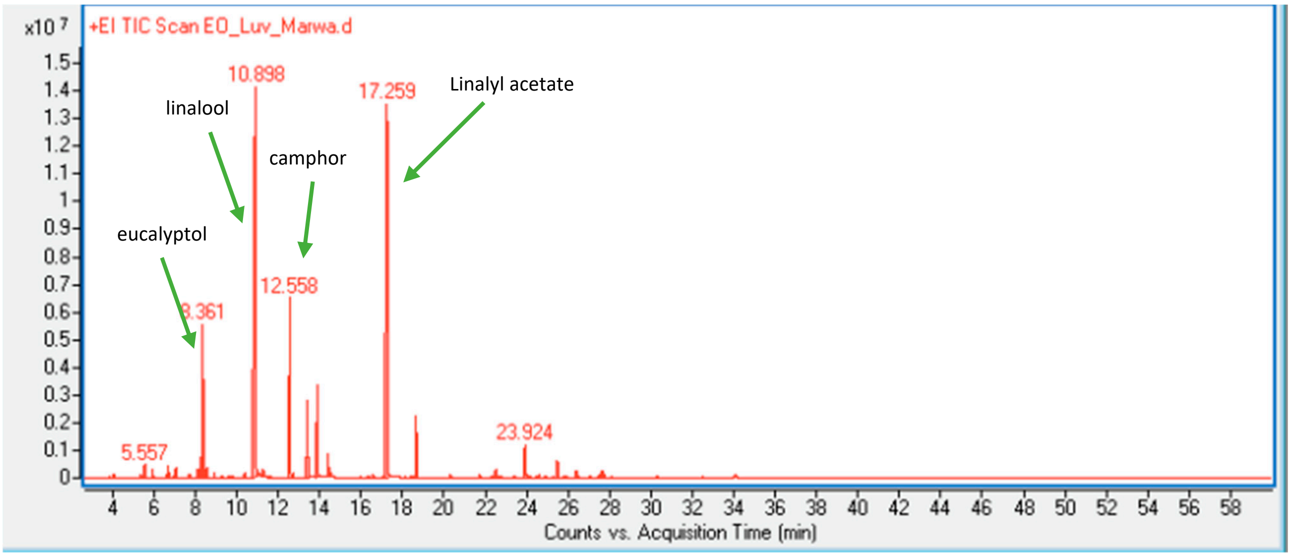

Figure S3. Chromatogram of *Lavandula hybrida* EO

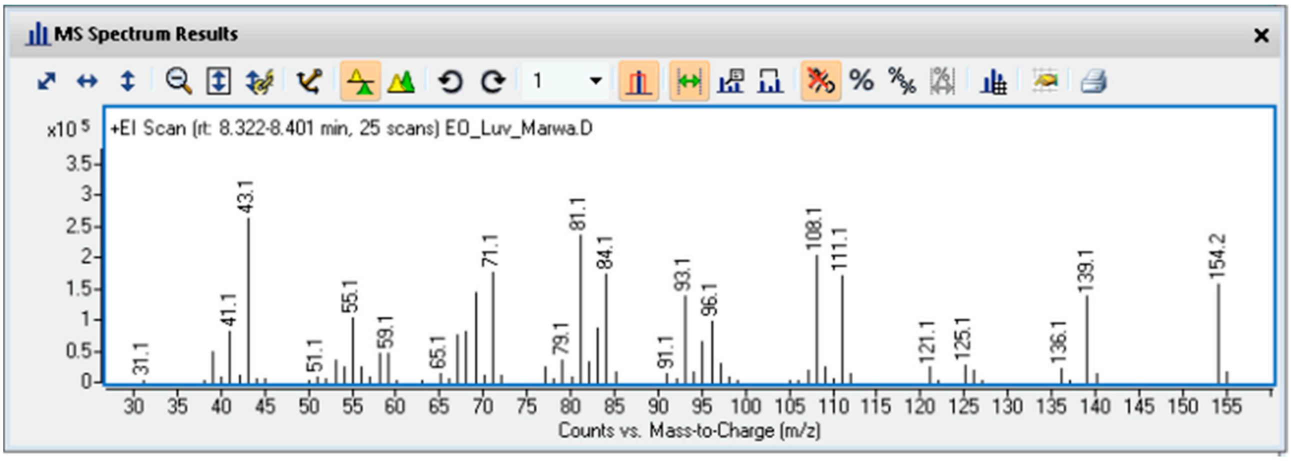

Figure S3. a. MS spectrum of eucalyptol

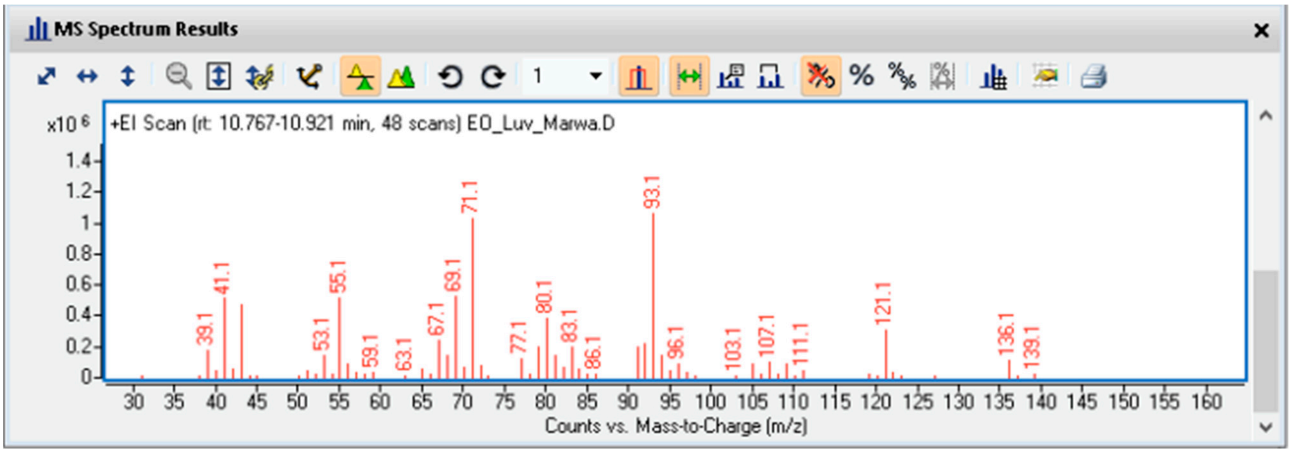

Figure S3. b. MS spectrum of linalool

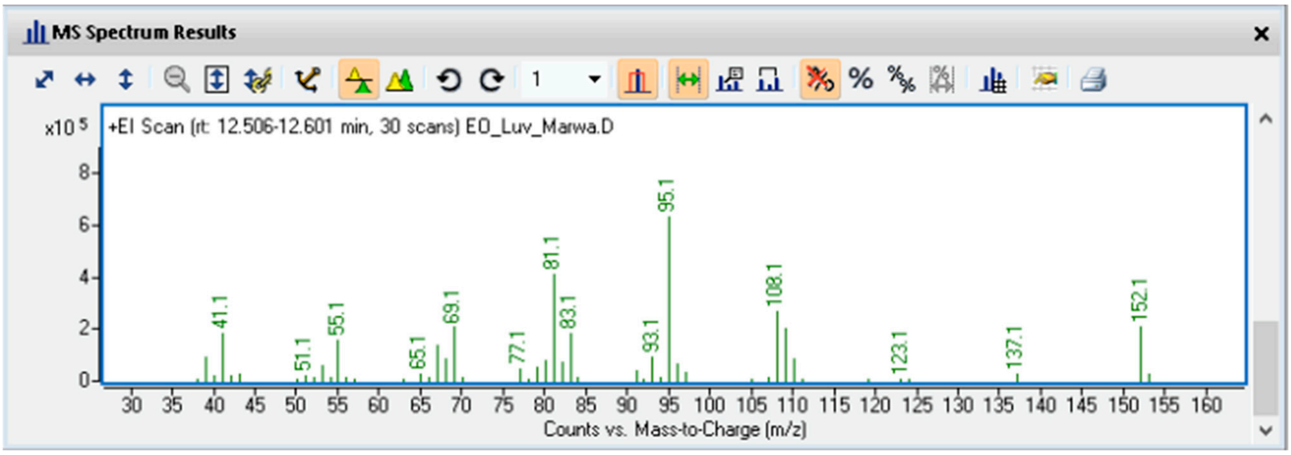

Figure S3. c. MS spectrum of camphor

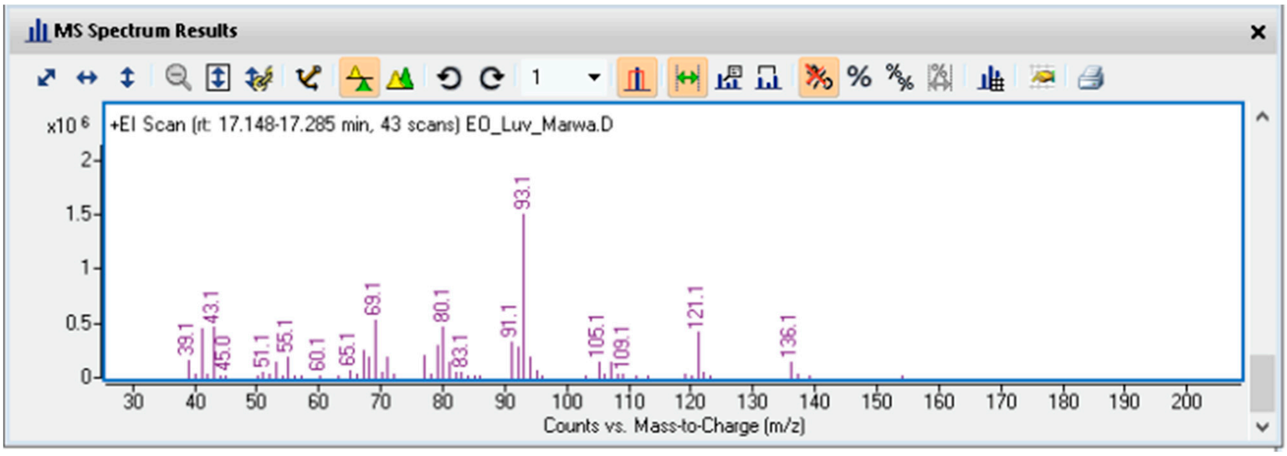

Figure S3. d. MS spectrum of linalyl acetate

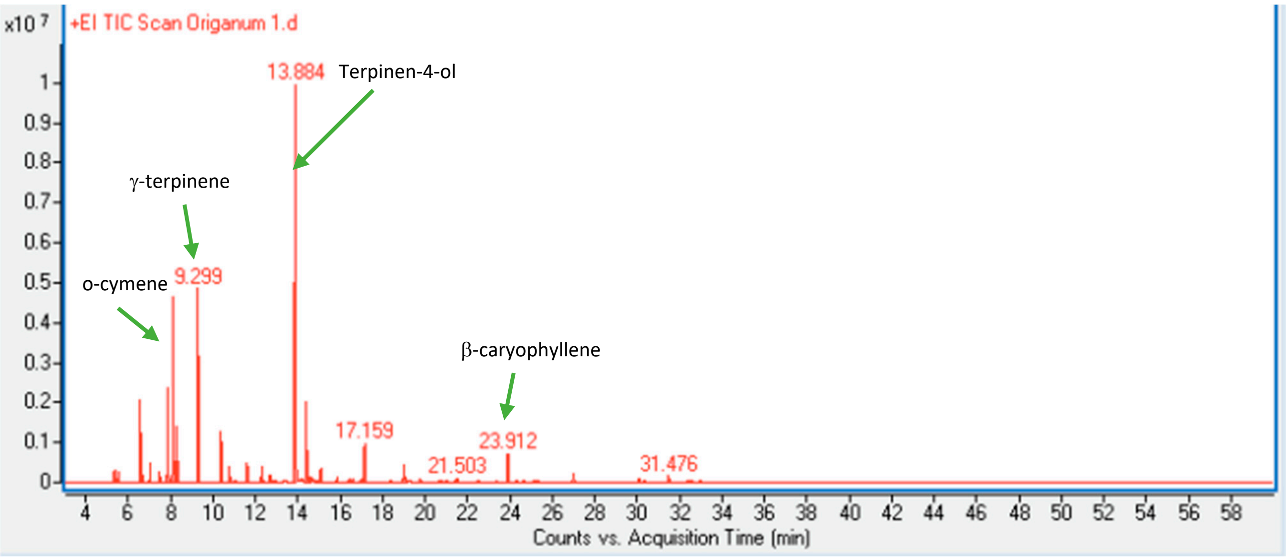

Figure S4. Chromatogram of *Origanum majorana*1 EO

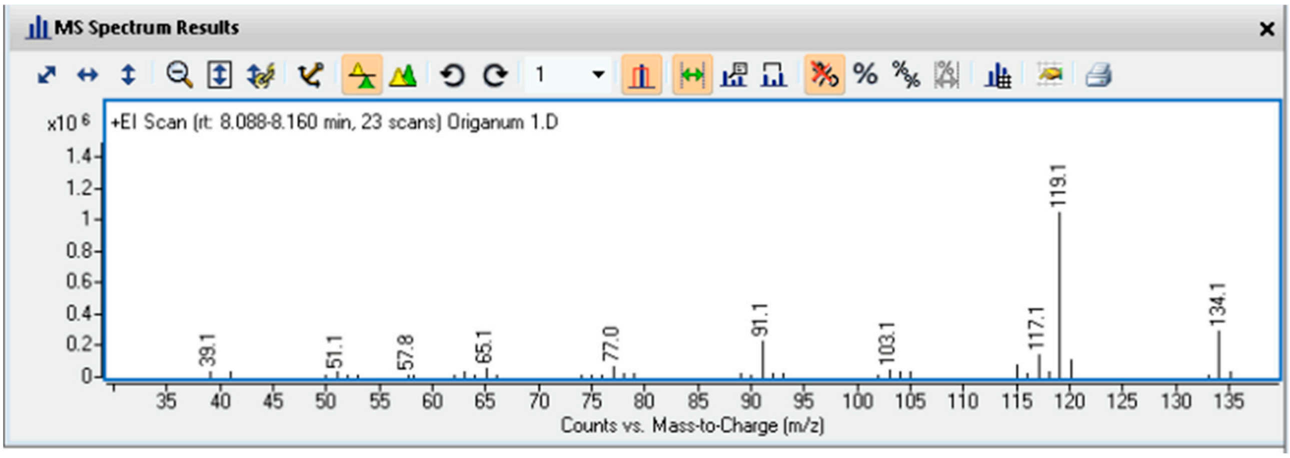

Figure S4. a. MS spectrum of o-cymene

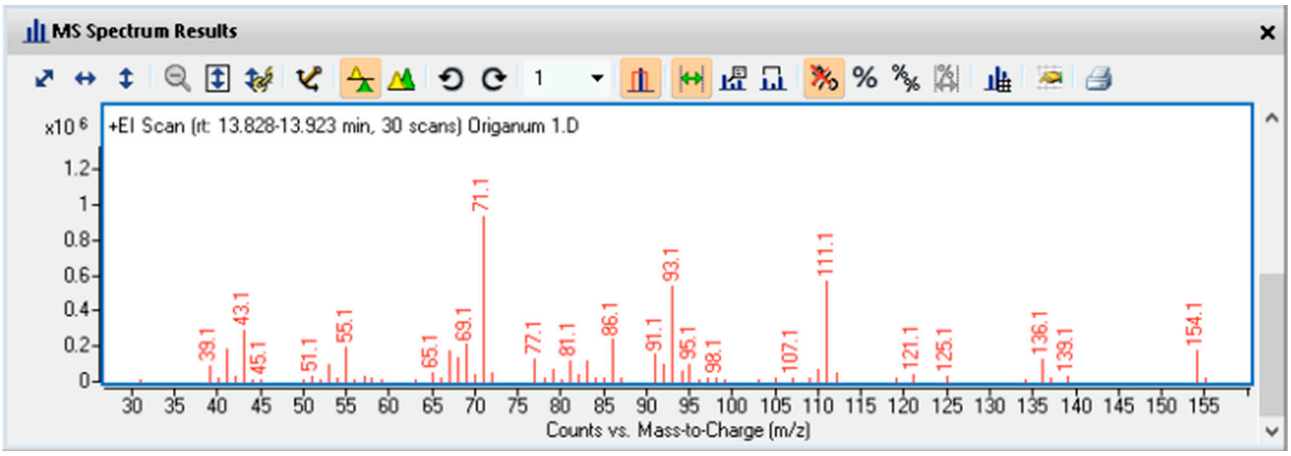

Figure S4. b. MS spectrum of terpinene-4-ol

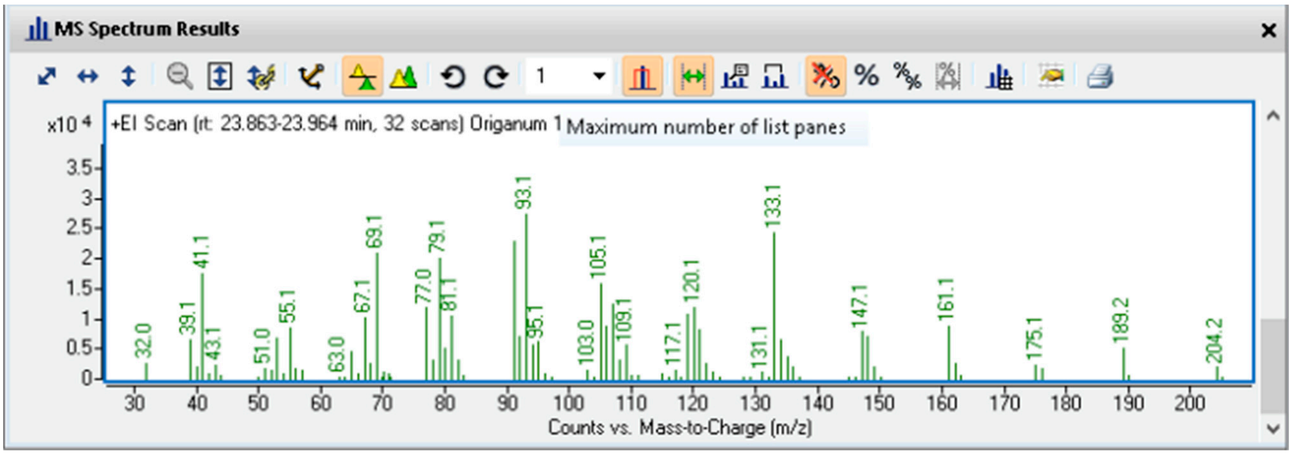

Figure S4. c. MS spectrum of b-caryophyllene

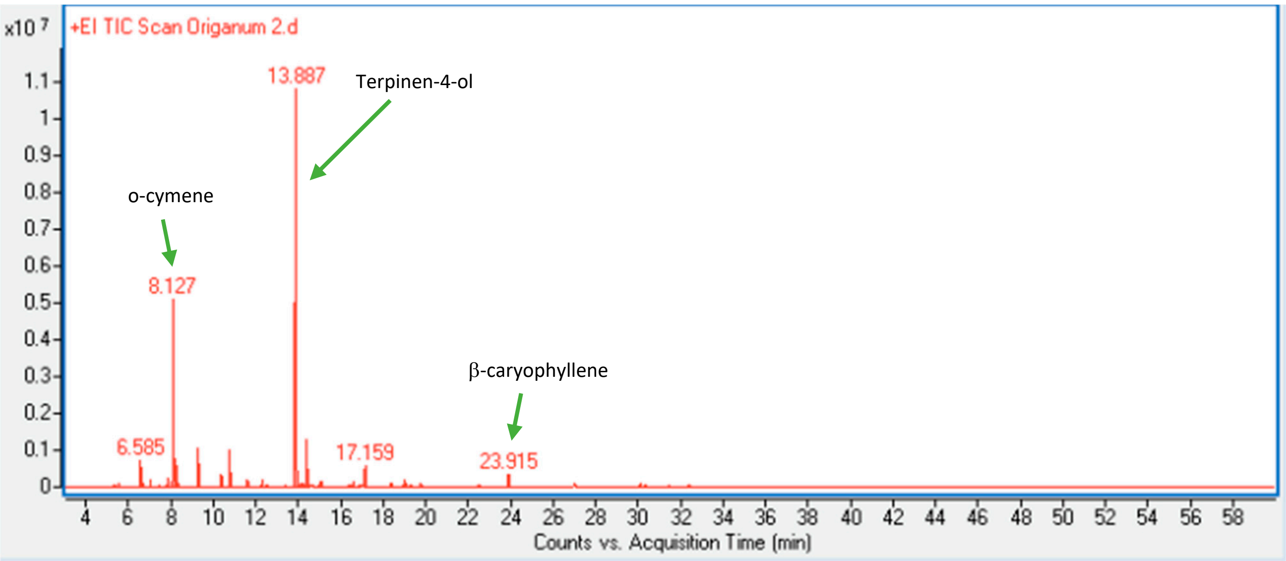

Figure S5. Chromatogram of *Origanum majorana*2 EO

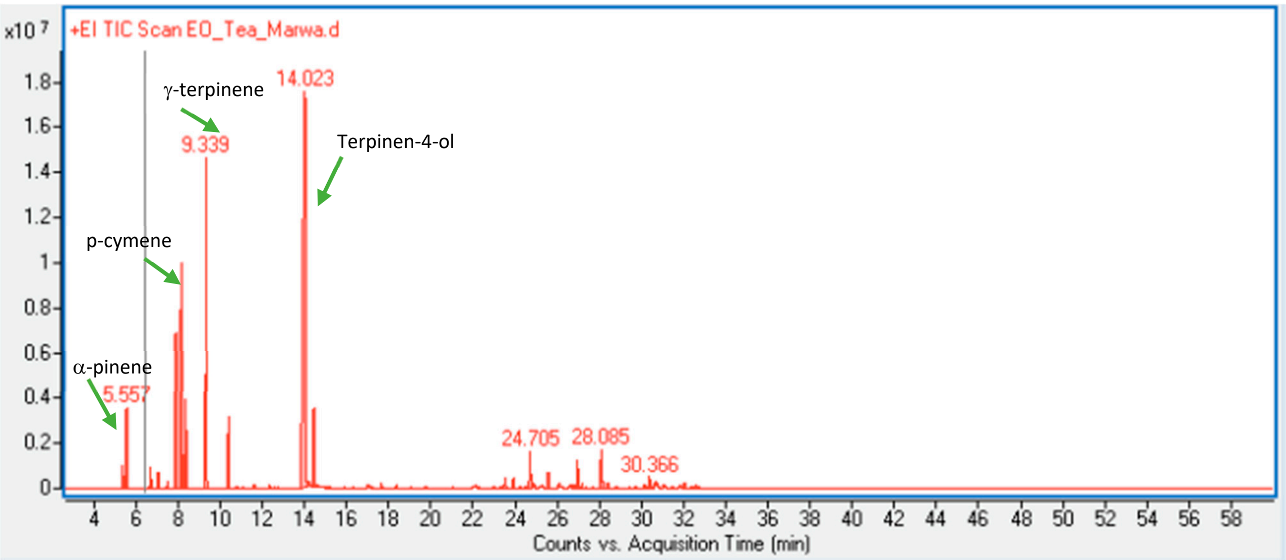

Figure S6. Chromatogram of *Melaleuca alternifolia* EO

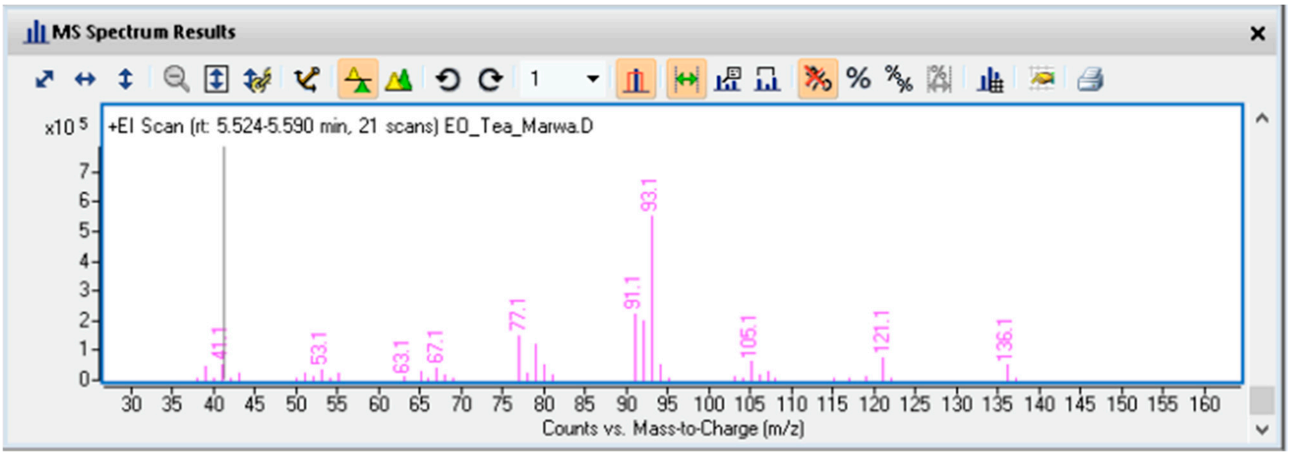

Figure S6. a. MS spectrum of a-pinene

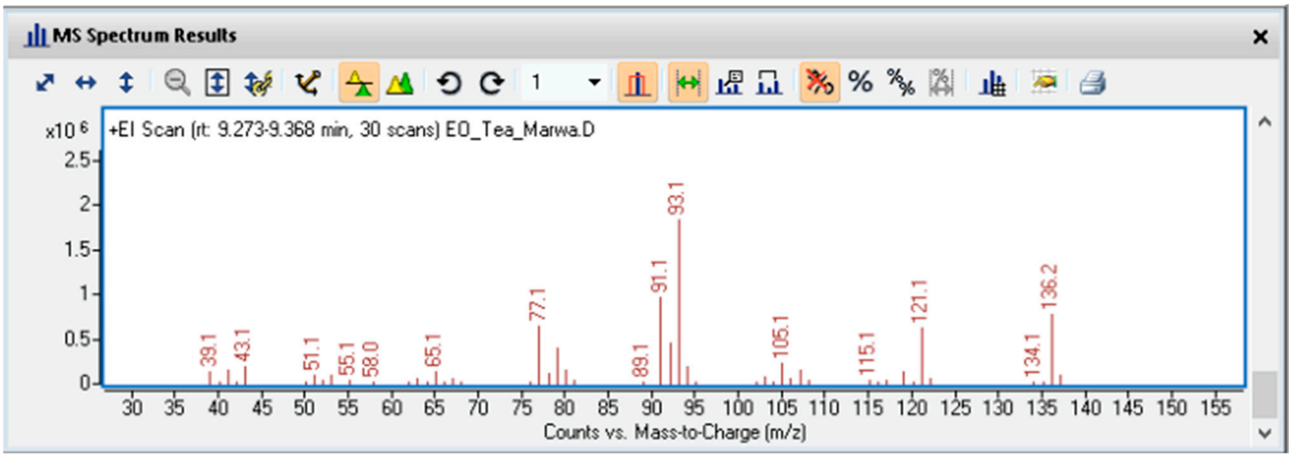

Figure S6. b. MS spectrum of g-terpinene

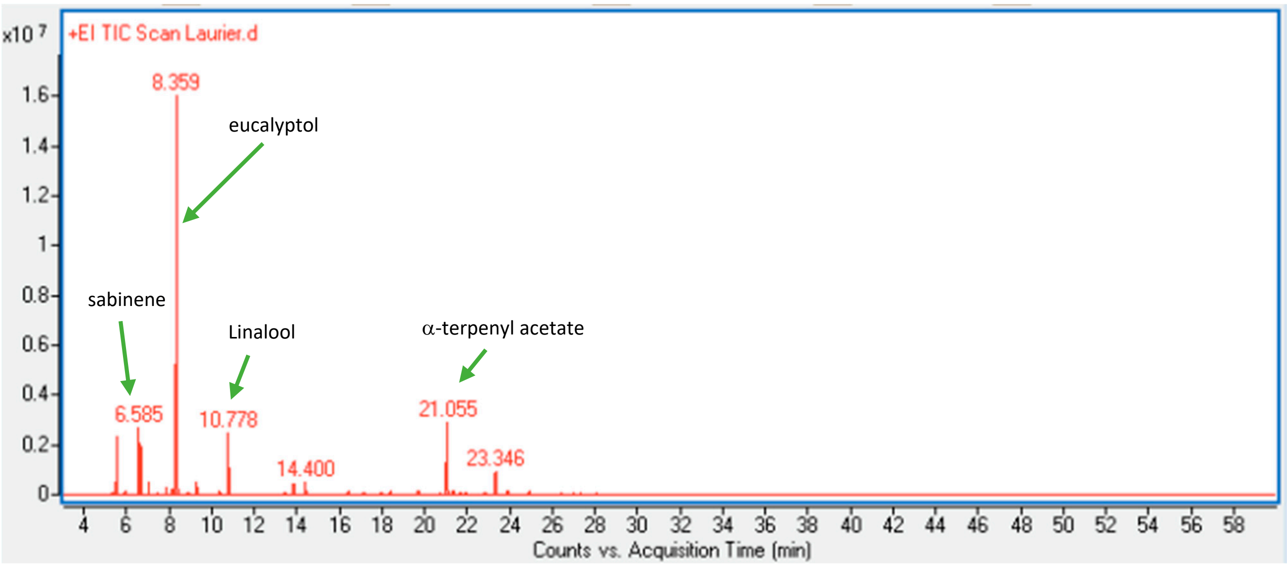

Figure S7. Chromatogram of *Laurus nobilis* EO

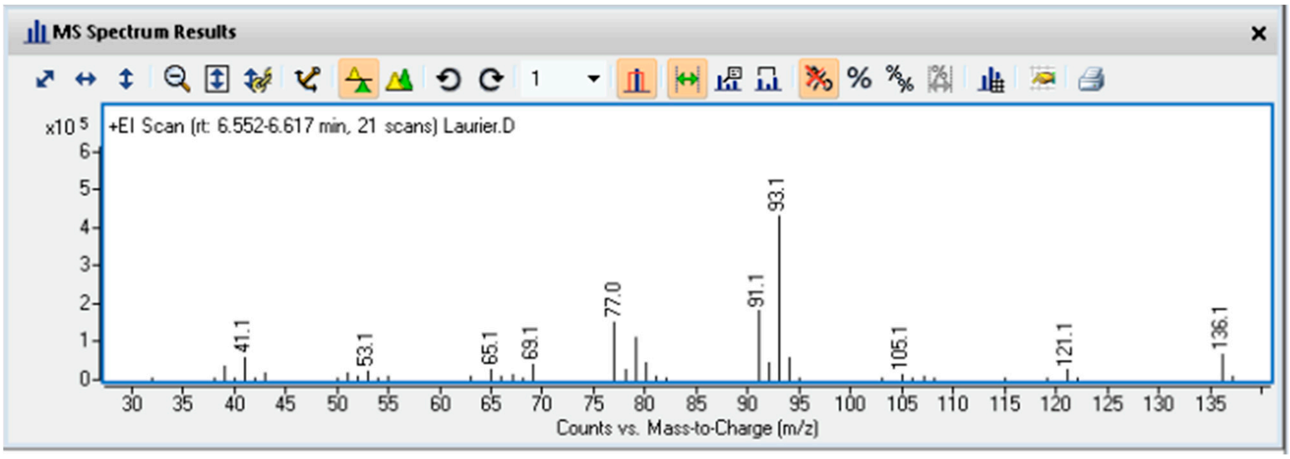

Figure S7. a. MS spectrum of sabinene

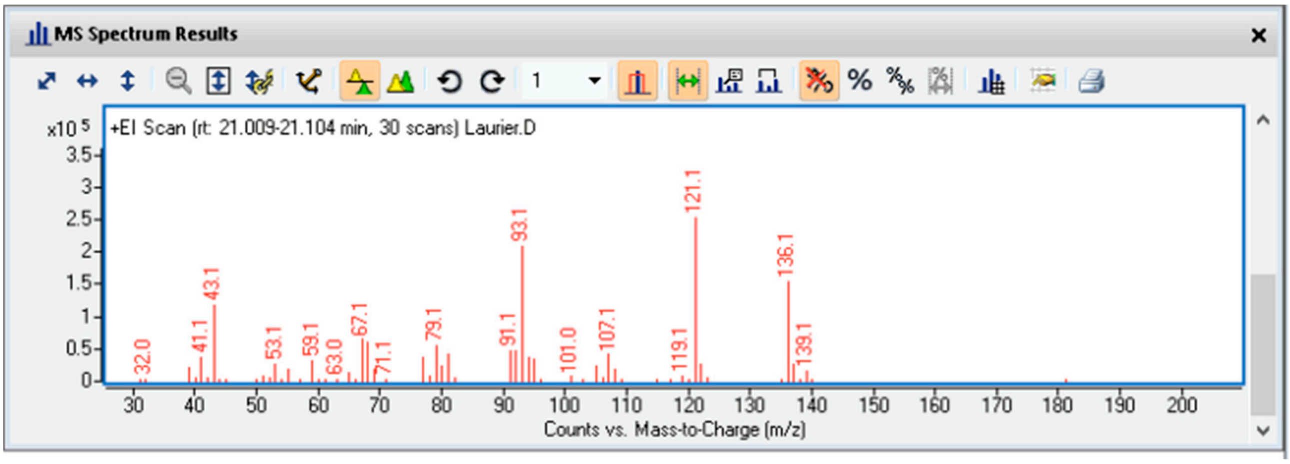

Figure S7. b. MS spectrum of a-terpenyl acetate
